# Association of dietary adherence and dietary quality with weight loss success among those following low-carbohydrate and low-fat diets: a secondary analysis of the DIETFITS randomized clinical trial

**DOI:** 10.1016/j.ajcnut.2023.10.028

**Published:** 2023-11-04

**Authors:** Michelle E Hauser, Jennifer C Hartle, Matthew J Landry, Priya Fielding-Singh, Cynthia W Shih, FeiFei Qin, Joseph Rigdon, Christopher D Gardner

**Affiliations:** 1 Stanford Prevention Research Center, Department of Medicine, Stanford University School of Medicine, Stanford, CA, United States; 2 Division of General Surgery, Department of Surgery, Stanford University School of Medicine, Stanford, CA, United States; 3 Division of Primary Care and Population Health, Department of Medicine, Stanford University, Stanford, CA, United States; 4 Internal Medicine-Obesity Medicine, Palo Alto Veterans Affairs Health Care System, Palo Alto, CA, United States; 5 Department of Public Health and Recreation, San José State University, San José, CA, United States; 6 Department of Family and Consumer Studies, University of Utah, Salt Lake City, UT, United States; 7 Quantitative Sciences Unit, School of Medicine, Stanford University, Stanford CA, United States; 8 Department of Biostatistics and Data Science, Wake Forest School of Medicine, Winston-Salem, NC, United States

**Keywords:** diet quality, low-carbohydrate, low-fat, weight loss, Healthy Eating Index

## Abstract

**Background:**

Eating a high-quality diet or adhering to a given dietary strategy may influence weight loss. However, these 2 factors have not been examined concurrently for those following macronutrient-limiting diets.

**Objective:**

To determine whether improvement in dietary quality, change in dietary macronutrient composition, or the combination of these factors is associated with differential weight loss when following a healthy low-carbohydrate (HLC) or healthy low-fat (HLF) diet.

**Design:**

Generally healthy adults were randomly assigned to HLC or HLF diets for 12 mo (n = 609) as part of a randomized controlled weight loss study. Participants with complete 24-h dietary recall data at baseline and 12-mo were included in this secondary analysis (total *N =* 448; *N =* 224 HLC, *N =* 224 HLF). Participants were divided into 4 subgroups according to 12-mo change in HEI-2010 score [above median = high quality (HQ) and below median = low quality (LQ)] and 12-mo change in macronutrient intake [below median = high adherence (HA) and above median = low adherence (LA) for net carbohydrate (g) or fat (g) for HLC and HLF, respectively]. Baseline to 12-mo changes in mean BMI were compared for those in HQ/HA, HQ/LA, LQ/HA subgroups with the LQ/LA subgroup within HLC and HLF.

**Results:**

For HLC, changes (95 % confidence level [CI]) in mean BMI were -1.15 kg/m^2^ (-2.04, -0.26) for HQ/HA, -0.30 (-1.22, 0.61) for HQ/LA, and -0.80 (-1.74, 0.14) for LQ/HA compared with the LQ/LA subgroup. For HLF, changes (95% CI) in mean BMI were -1.11kg/m^2^ (-2.10, -0.11) for HQ/HA, -0.26 (-1.26, 0.75) for HQ/LA, and -0.66 (-1.74, 0.41) for LQ/HA compared with the LQ/LA subgroup.

**Conclusion:**

Within both HLC and HLF diet arms, 12-mo decrease in BMI was significantly greater in HQ/HA subgroups relative to LQ/LA subgroups. Neither HQ nor HA alone were significantly different than LQ/LA subgroups. Results of this analysis support the combination of dietary adherence and high-quality diets for weight loss.

**Clinical Trial Registry:**

clinicaltrials.gov (Identifier: NCT01826591).

## Introduction

According to the Centers for Disease Control National Center for Health Statistics, 42.4% of adults in the United States had obesity in 2017 and 2018, and it is predicted that by 2030, nearly 1 in 2 adults will have obesity [1, 2]. Obesity, dietary risks, hypertension, elevated fasting plasma glucose, and hyperlipidemia make up 5 of the top 6 risk factors for early mortality in the United States [3]. Dietary modification via a variety of healthy diet approaches has been shown to improve cardiometabolic risk factors and reduce weight [4, 5]. Therefore, it has long been a cornerstone of most successful weight loss strategies both in research and practice. Low-carbohydrate (LC) and low-fat diets (LF) are among the most studied approaches, but neither has been shown to be consistently superior for weight loss in the general population [[5], [6], [7], [8], [9], [10]]. Previous studies suggest that eating a high-quality diet or adhering to a given dietary strategy may influence weight loss; however, these 2 factors have not been examined concurrently for those following macronutrient-limiting diets.

Although evidence supports the use of LC or LF diets in weight loss efforts [5], the benefits of simultaneously focusing on achieving higher quality diets are unclear. To determine whether improvement in dietary quality, change in dietary macronutrient composition, or the combination of these factors associated with differential weight loss and change in cardiovascular disease risk factors, a post hoc secondary analysis was conducted using data from the Diet Intervention Examining The Factors Interacting with Treatment Success (DIETFITS) weight loss trial [11] in which randomly assigned participants follow a healthy low-carbohydrate (HLC) or healthy low-fat (HLF) diet for 12 mo. The dataset presented a unique opportunity because the intervention dually emphasized both reduction in macronutrient intake and improvement in dietary quality [12], whereas the majority of LC versus LF weight loss studies primarily focuses on macronutrient reduction [8, 10, [13], [14], [15], [16], [17], [18], [19]].

In this exploratory analysis, we aimed to determine whether those who most improved the quality of their diet, those who most decreased their net carbohydrate or fat intake, or both were more successful in their weight loss endeavors than those who did not improve their dietary quality nor reduce their assigned macronutrient intake to the same degree when assigned to follow HLC or HLF diets. Secondarily, we aimed to determine the impact of these factors on changes in cardiovascular disease risk factors, including blood pressure, fasting insulin, glucose, and lipids.

## Methods

### Study design

Our post hoc secondary analysis utilized data from the DIETFITS study, of which detailed methods have previously been published [12]. In brief, this was a single-site, parallel-group, randomized controlled weight loss trial of 609 free-living, generally healthy males and premenopausal females aged 18 to 50 y, with BMI from 28 to 40 kg/m^2^, who were randomly assigned to HLC or HLF diets for 12 mo to determine whether genetics (via 3-single nucleotide polymorphisms) or metabolic predispositions (insulin secretion) at baseline resulted in differential weight loss for those assigned to either diet. Randomization was performed using an allocation sequence determined by computerized random-number generation (Blockrand in R version 3.4.0, R Project for Statistical Computing) in block sizes of 8 (with 4 individuals going to each diet group) by a statistician not involved in intervention delivery or data collection. The trial enrollment was from January 29, 2013 through April 14, 2015, and the data of the final follow-up was from May 16, 2016. The study was conducted in the San Francisco Bay Area of California. Key exclusion criteria included—pregnancy or nursing, uncontrolled metabolic disease, diabetes, cancer, liver, kidney, or heart disease, and taking psychiatric medications or medications known to affect weight or energy expenditure, serum lipids, serum glucose, or blood pressure.

The DIETFITS trial was overseen by the Stanford University Institutional Review Board and was registered with clinicaltrials.gov (Identifier: NCT01826591). All study participants provided written informed consent. All data from the DIETFITS randomized trial were managed using the REDCap electronic data capture tool hosted at Stanford University [20].

After baseline data collection, participants’ random assignment to the HLC or HLF diet was revealed at their first intervention class. The intervention consisted of 22 sessions of class-based instruction conducted by registered dietitian nutritionists (RDNs) held over 12 mo within diet groups. Classes were conducted weekly for 8 wk, then every 2 wk for 2 mo, then every 3 wk until Month 6, and then monthly through Month 12. Dietary goals included reducing net carbohydrate or fat intake to 20 g or less for 8 wk, then slowly increasing over time until each participant found the level of intake they could sustain indefinitely, reducing intake of highly processed foods and added sugars, and maximizing intake of vegetables and other whole foods. There was no explicit energy restriction. Dietary data collection was conducted via 3 unannounced, 24-h dietary recalls using a standardized, multiple-pass approach [21] that included 2 weekdays and 1 weekend day at the data collection time points of baseline, 3, 6, and 12 mo.

For the current analysis, participants who provided complete 24-h dietary recall data at baseline and 12 mo were included (total *N =* 448; *N =* 224 HLC, *N =* 224 HLF) (Supplemental Figure 1). Diet quality scores were calculated from each dietary recall using the Healthy Eating Index-2010 (HEI-2010) [[22], [23], [24], [25]]. Then, scores from the 3 recalls from each time point for each participant were averaged to yield a single score per participant for each time point. Within each diet assignment arm, participants were divided into 4 subgroups according to 12-mo change in diet quality score [HEI-2010, above the median was defined as high quality (HQ); below the median was defined as low quality (LQ)] and 12-mo change in macronutrient intake [below the median was defined as high adherence (HA) and above the median was defined as low adherence (LA) for net carbohydrate (g) or fat (g) for HLC and HLF, respectively]. For the low-carbohydrate diet, the cutoff used for quality was 4.2 (median 12-mo change in HEI-2010), and the cutoff used for adherence was -103.2 (g) (median 12-mo change in carbohydrate). For the low-fat diet, the cutoff used for quality was 8.5 (median 12-mo HEI-2010), and the cutoff used for adherence was -29.7(g) (median 12-mo change in fat). Median values for 12-mo change in HEI-2010 and macronutrient intake by diet assignment are provided in Supplemental Tables 1 and 2. Therefore, the 4 subgroups for each diet were HQ/HA, HQ/LA, LQ/HA, and LQ/LA.

This study was a post hoc secondary analysis. The predefined primary outcome was 12-mo change in BMI. Predefined secondary outcomes included 12-mo change in the following cardiometabolic measures: systolic blood pressure (SBP), diastolic blood pressure (DBP), fasting plasma glucose, insulin, triglycerides, HDL cholesterol, and LDL cholesterol concentrations. The reference subgroup was LQ/LA. All clinical measurements (body weight, height, blood pressure, and a fasting blood draw, which would be analyzed for insulin, glucose, lipids, and triglycerides concentrations) were collected by the Stanford Clinical Translational Research Unit at each data collection time point [12].

### Statistical analysis

Descriptive statistics were used to display baseline demographic, clinical characteristics, and dietary components for each subgroup (Table 1 and Table 2). Descriptive statistics, including mean and standard deviation, were calculated for each of the primary and secondary outcomes and dietary components for each subgroup for all participants with data available (Table 3 and Table 4). Two multiple linear regression models were used to compare the primary and secondary outcomes for those in the HQ/HA, HQ/LA, LQ/HA subgroups versus the LQ/LA subgroup within HLC and HLF diet groups (Table 5). Model 1 was adjusted for age, sex, and baseline weight, whereas Model 2 additionally adjusted for either baseline net carbohydrate or fat intake for the HLC and HLF diet groups, respectively. Model 2 represents the main analyses for study primary and secondary outcomes. To ensure results were not related to baseline energy intake or HEI-2010 total score, we also added these factors to Model 2 (Supplemental Table 5). No formal between-diet statistical comparisons were conducted, given likely bias of diet quality indices toward low-fat versus low-carbohydrate diets (manuscript concurrently under review), although confidence intervals are provided. There was no correction for multiple testing as this was an exploratory, post hoc secondary analysis.

**TABLE 1:** Baseline characteristics by dietary quality and adherence to diet for those assigned to a healthy low-carbohydrate diet (*n =* 304).^1^^,^^2^^,^^3^

Variable	High quality, high adherence (HQ/HA) *N =* 67	High quality, low adherence (HQ/LA) *N =* 45	Low quality, high adherence (LQ/HA) *N =* 45	Low quality, low adherence (LQ/LA) *N =* 67	Total *N =* 224	Missing 12-mo dietary data *N =* 80
Sex (*N*, %)						
Female	42 (62.7)	27 (60.0)	24 (53.3)	39 (58.2)	132 (58.9)	47 (58.8)
Male	25 (37.3)	18 (40.0)	21 (46.7)	28 (41.8)	92 (41.1)	33 (41.2)
Age (y)	41.22 (6.15)	40.05 (6.58)	41.33 (7.04)	39.98 (6.80)	40.6 (6.6)	38.98 (6.97)
Education (*N*, %)						
Less than high school	0 (0.0)	2 (4.4)	0 (0.0)	1 (1.5)	3 (1.3)	1 (1.2)
High school graduate	0 (0.0)	1 (2.2)	1 (2.2)	3 (4.5)	5 (2.2)	6 (7.5)
Some college	12 (17.9)	7 (15.6)	4 (8.9)	18 (26.9)	41 (18.3)	26 (32.5)
College graduate	33 (49.3)	20 (44.4)	22 (48.9)	20 (29.9)	95 (42.4)	23 (28.7)
Postgraduate degree	22 (32.8)	15 (33.3)	18 (40.0)	25 (37.3)	80 (35.7)	23 (28.7)
Race and ethnicity (*N*, %)^4^						
White	43 (64.2)	24 (53.3)	32 (71.1)	39 (58.2)	138 (61.6)	44 (55.0)
Hispanic	12 (17.9)	9 (20.0)	8 (17.8)	13 (19.4)	42 (18.8)	19 (23.8)
Asian	7 (10.4)	8 (17.8)	2 (4.4)	7 (10.4)	24 (10.7)	6 (7.5)
African American	0 (0.0)	1 (2.2)	3 (6.7)	5 (7.5)	9 (4.0)	4 (5.0)
Other^5^	5 (7.5)	3 (6.7)	0 (0.0)	3 (4.5)	11 (4.9)	7 (8.8)
Weight (kg)						
Female	88.5 (12.2)	86.5 (12.4)	89.2 (12.1)	86.8 (12.2)	87.7 (12.1)	92.3 (13.0)
Male	104.5 (12.8)	110.7 (17.3)	104.6 (9.4)	104.6 (16.3)	105.8 (14.3)	109.5 (11.8)
BMI (kg/m^2^)	33.22 (3.14)	33.58 (3.55)	33.19 (3.50)	32.43 (3.36)	33.1 (3.4)	34.2 (3.5)
LDL (mg/dL)	118.25 (26.77)	114.80 (22.74)	118.55 (28.13)	114.91 (28.58)	116.6 (26.7)	108.9 (24.8)
HDL (mg/dL)						
Female	52.0 (11.2)	52.1 (9.5)	49.1 (10.7)	50.2 (9.0)	51.0 (10.1)	51.1 (11.7)
Male	40.6 (7.4)	43.0 (9.0)	46.3 (9.1)	45.2 (9.7)	43.8 (9.0)	42.5 (10.0)
Triglycerides (mg/dL)	142.40 (71.61)	123.14 (49.94)	150.23 (225.37)	123.78 (56.11)	134.5 (114.4)	116.8 (53.8)
SBP (mmHg)	122.97 (12.99)	122.39 (12.63)	122.32 (11.15)	123.40 (12.80)	122.9 (12.4)	123.3 (12.5)
DBP (mmHg)	81.07 (8.69)	80.84 (8.18)	81.13 (6.37)	81.43 (8.19)	81.2 (8.0)	81.6 (7.7)
Fasting insulin (μU/mL)	16.88 (6.51)	15.40 (6.14)	13.35 (4.81)	13.71 (7.72)	14.9 (6.7)	16.9 (10.9)
Fasting glucose (mL/dL)	100.54 (11.34)	96.53 (7.45)	96.27 (8.75)	97.87 (10.77)	98.1 (10.1)	98.8 (8.8)
Waist circumference (cm)						
Female	104.1 (9.1)	100.4 (13.4)	102.1 (7.9)	98.7 (9.9)	101.4 (10.3)	105.9 (10.6)
Male	113.2 (8.1)	114.8 (11.0)	109.9 (6.7)	110.9 (11.2)	112.1 (9.5)	114.4 (11.0)
Baseline Dietary Components						
Total Energy (kcal)	2490.05 (622.57)	1843.27 (469.64)	2451.06 (631.52)	2024.14 (578.05)	2212.93 (639.23)	2251.52 (693.38)
Intake of Carbohydrate (g)	263.85 (68.14)	181.19 (53.89)	246.98 (62.71)	195.1 (63.98)	223.29 (71.68)	229.70 (80.80)
Intake of Fat (g)	101.14 (32.32)	78.05 (24.99)	100.99 (36.24)	86.34 (32.5)	92.04 (33.08)	94.09 (35.02)
HEI-2010 Score	48.7 (9.9)	51.22 (10.87)	61.88 (11.99)	61.26 (9.91)	55.61 (12.06)	53.10 (11.30)
12-mo dietary components						---
Total energy (kcal)	1581.38 (400.27)	1745.44 (500.43)	1589.74 (428.66)	1852.35 (531.05)	1697.07 (480.22	---
Intake of carbohydrate (g)	90.25 (37.76)	132.9 (53.67)	84.46 (51.19)	144.27 (69.49)	113.81 (60.25)	---
Intake of fat (g)	85.47 (29.87)	86.22 (29.02)	86.04 (28.38)	87.13 (32.2)	86.23 (29.94)	---
HEI-2010 Score	63.86 (8.78)	64.61 (10.8)	56.54 (11.35)	54.81 (9.48)	59.83 (10.82)	---

Abbreviations: SBP, Systolic Blood Pressure; DBP, Diastolic Blood Pressure; HEI, Healthy Eating Index -2010.

1 Dietary quality is defined by 12-mo minus baseline change in HEI-2010 score (above the median is high, below the median is low).

2 Dietary adherence is defined by 12-mo minus baseline change in grams of carbohydrate (below the median is high, above the median is low).

3 All values presented as Mean (SD) unless specified.

4 Self-reported by participants from the following options (White, Hispanic, Black, Asian, American Indian, Alaska Native, Pacific Islander and Other).

5 American Indian/Alaska Native/Pacific Islander and Other.

**TABLE 2:** Baseline characteristics by dietary quality and adherence to diet for those assigned to a healthy low-fat diet (*n =* 305)^1^^,^^2^^,^^3^.

	High quality, high adherence (HQ/HA) *N =* 64	High quality, low adherence (HQ/LA) *N =* 48	Low quality, high adherence (LQ/HA) *N =* 48	Low quality, low adherence (LQ/LA) *N =* 64	Total *N =* 224	Missing 12-mo dietary data *N =* 81
Sex (*N*, %)						
Female	38 (59.4)	33 (68.8)	18 (37.5)	37 (57.8)	126 (56.2)	41 (50.6)
Male	26 (40.6)	15 (31.3)	30 (62.5)	27 (42.2)	98 (43.8)	40 (49.4)
Age (y)	39.9 (7.3)	40.4 (6.0)	40.3 (6.1)	39.3 (6.7)	39.94 (6.57)	37.72 (7.17)
Education (*N*, %)						
Less than college degree	15 (23.5)	12 (25.0)	2 (4.2)	17 (26.6)	46 (20.6)	24 (29.6)
College graduate	24 (37.5)	13 (27.1)	30 (62.5)	22 (34.4)	89 (39.7)	38 (46.9)
Postgraduate degree	24 (37.5)	23 (47.9)	16 (33.3)	25 (39.1)	88 (39.3)	19 (23.5)
Race and ethnicity (*N*, %)^4^						
White	42 (65.6)	24 (50.0)	33 (68.8)	35 (54.7)	134 (59.8)	42 (51.9)
Hispanic	11 (17.2)	16 (33.3)	6 (12.5)	12 (18.8)	45 (20.1)	22 (27.2)
Asian	5 (7.8)	4 (8.3)	5 (10.4)	7 (10.9)	21 (9.4)	9 (11.1)
African American	3 (4.7)	2 (4.2)	0 (0.0)	3 (4.7)	8 (3.6)	2 (2.5)
Other^5^	3 (4.7)	2 (4.2)	4 (8.3)	7 (10.9)	16 (7.1)	6 (7.4)
Weight (kg)						
Female	93.2 (10.3)	87.5 (9.2)	91.6 (13.6)	90.0 (13.4)	90.6 (11.6)	91.1 (11.1)
Male	107.1 (17.1)	105.2 (12.2)	106.5 (10.9)	101.9 (11.1)	105.2 (13.0)	107.1 (15.8)
BMI (kg/m^2^)	33.9 (3.2)	33.0 (3.1)	33.9 (2.7)	33.0 (3.6)	33.45 (3.20)	33.36 (3.41)
LDL (mg/dL)	114.1 (30.7)	106.6 (26.9)	108.0 (33.1)	117.7 (33.1)	112.22 (31.28)	114.19 (29.60)
HDL (mg/dL)						
Female	49.9 (9.2)	50.5 (11.5)	53.9 (18.4)	50.4 (9.7)	50.8 (11.6)	50.7 (9.6)
Male	43.3 (7.8)	44.7 (9.0)	43.0 (12.2)	40.2 (7.5)	42.6 (9.4)	45.1 (7.4)
Triglycerides (mg/dL)	138.3 (75.8)	127.9 (101.4)	131.5 (65.6)	130.6 (71.0)	132.4 (78.3)	124.4 (55.8)
SBP (mmHg)	124.1 (12.2)	124.3 (14.5)	127.5 (14.0)	119.9 (12.4)	123.7 (13.4)	121.5 (11.1)
DBP (mmHg)	81.8 (6.6)	82.0 (8.4)	84.4 (9.1)	78.8 (6.5)	81.5 (7.8)	80.3 (6.6)
Fasting insulin (μU/mL)	15.8 (8.0)	15.1 (11.5)	16.4 (7.6)	17.4 (24.8)	16.2 (15.3)	14.8 (6.4)
Fasting glucose (mL/dL)	99.3 (8.2)	97.8 (9.6)	98.6 (8.7)	97.9 (9.0)	98.4 (8.8)	98.6 (8.1)
Waist circumference (cm)						
Female	104.5 (9.2)	103.5 (13.3)	104.9 (9.6)	103.2 (10.8)	103.9 (10.8)	102.3 (9.2)
Male	112.5 (11.6)	111.6 (8.3)	114.3 (8.4)	109.0 (8.4)	112.0 (9.4)	111.4 (10.5)
Dietary Components						
Total Energy (kcal)	2555.5 (661.54)	1686.41 (457.73)	2560.23 (461)	1950.03 (566.82)	2197.29 (665.02)	2010.24 (732.12)
Intake of Carbohydrate (g)	264.24 (89.9)	181.43 (68.05)	249.76 (71.21)	205.19 (62.85)	226.52 (80.85)	200.99 (79.61)
Intake of Fat (g)	107.54 (31.38)	62.51 (22.62)	111.96 (26.54)	73.9 (28.07)	89.22 (34.43)	80.88 (35.11)
HEI-2010 Score	52.82 (10.92)	53.51 (10.43)	58.79 (9.17)	61.23 (10.95)	56.65 (11.02)	52.40 (12.63)
12-mo Dietary Components						—
Total Energy (kcal)	1621.24 (450.59)	1554.29 (472.19)	1756.26 (460.89)	1914.07 (588.09)	1719.49 (516.36)	—
Intake of Carbohydrate (g)	189.44 (67.22)	162.83 (67.04)	199.6 (64.86)	204.38 (72.38)	190.19 (69.49)	—
Intake of Fat (g)	47.95 (19.03)	55.3 (24.56)	55.54 (22.33)	70.2 (27.81)	57.51 (25.05)	—
HEI-2010 Score	69.9 (10.76)	71.15 (8.97)	57.68 (8.88)	57.31 (12.23)	63.95 (12.29)	—

Abbreviations: SBP, Systolic Blood Pressure; DBP, Diastolic Blood Pressure; HEI, Healthy Eating Index -2010.

1 Dietary quality is defined by 12-mo minus baseline change in HEI-2010 score (above the median is high, below the median is low).

2 Dietary adherence is defined by 12-mo minus baseline change in grams of fat (below the median is high, above the median is low).

3 All values presented as Mean (SD) unless specified.

4 Self-reported by participants from the following options (White, Hispanic, Black, Asian, American Indian, Alaska Native, Pacific Islander and Other).

5 American Indian/Alaska Native/Pacific Islander and Other.

**TABLE 3:** Baseline to 12-mo change in primary and secondary outcome variables by dietary quality and adherence for those assigned to a healthy low-carbohydrate diet^1^^,^^2^^,^^3^.

	*N*	High quality, high adherence *N =* 67	*N*	High quality, low adherence *N =* 45	*N*	Low quality, high adherence *N =* 45	*N*	Low quality, low adherence *N =* 67	*N*	Total *N =* 224	*N*	Missing *N =* 80
Primary outcome												
BMI (kg/m^2^)	62	-2.49 (2.33)	42	-2.22 (2.84)	44	-2.25 (2.11)	62	-1.73 (2.26)	210	-2.16 (2.38)	70	-2.33 (1.74)
Secondary outcomes												
Fasting glucose (mL/dL)	62	-4.31 (9.94)	42	-1.96 (8.54)	43	-1.38 (9.79)	62	0.26 (9.68)	209	-1.88 (9.66)	70	-4.60 (4.45)
Fasting insulin (μU/mL)	62	-3.87 (4.34)	42	-2.90 (5.69)	43	-2.05 (4.62)	62	0.05 (5.86)	210	-2.14 (5.36)	70	-1.95 (2.53)
SBP (mmHg)	62	-5.41 (12.52)	42	-4.19 (7.24)	44	-5.25 (7.78)	62	-0.56 (8.16)	210	-3.70 (9.61)	70	0.25 (5.27)
DBP (mmHg)	62	-3.57 (8.06)	42	-2.45 (5.06)	44	-3.61 (5.39)	62	-1.04 (5.11)	209	-2.61 (6.23)	70	-0.78 (3.75)
Triglycerides (mg/dL)	62	-40.77 (54.32)	42	-20.61 (48.50)	43	-52.73 (191.12)	62	-13.35 (36.78)	208	-31.05 (96.65)	70	-19.35 (37.21)
HDL-C (mg/dL)	62	3.19 (6.25)	42	0.92 (5.56)	42	2.76 (6.96)	62	3.81 (7.02)	208	2.83 (6.54)	70	-0.45 (5.29)
LDL-C (mg/dL)	62	5.39 (21.78)	42	4.17 (18.31)	42	2.34 (19.44)	62	2.08 (20.79)	224	3.54 (20.26)	70	-2.53 (19.43)
Dietary components												
Energy (kcal)	67	-908.67 (569.93)	45	-97.83 (381.11)	45	-861.32 (566.08)	67	-171.79 (496.66)	224	-515.86 (634.27)	-	-
Carbohydrate (g)	67	-173.60 (58.28)	45	-48.29 (36.51)	45	-162.53 (45.49)	67	-50.83 (43.27)	224	-109.48 (76.31)	-	-
Fat (g)	67	-15.66 (37.60)	45	8.18 (28.18)	45	-14.95 (43.61)	67	0.79 (37.00)	224	-5.81 (38.13)	-	-
HEI-2010 Score	67	15.16 (7.11)	45	13.39 (5.99)	45	-5.35 (8.78)	67	-6.45 (7.31)	224	4.22 (12.60)	-	-

Abbreviations: SBP, Systolic Blood Pressure; DBP, Diastolic Blood Pressure; HEI, Healthy Eating Index -2010.

1 Dietary quality is defined by 12-mo minus baseline change in HEI-2010 score (above the median is high, below the median is low).

2 Dietary adherence is defined by 12-mo minus baseline change in grams of carbohydrate (below the median is high, above the median is low).

3 All values presented as Mean (SD). *N* columns are the number of participants whose data was available to include in the analysis of each outcome.

**TABLE 4:** Baseline to 12-mo change in primary and secondary outcome variables by dietary quality and adherence for those assigned to a healthy low-fat diet^1^^,^^2^^,^^3^.

	*N*	High quality, high adherence *N =* 64	*N*	High quality, low adherence *N =* 48	*N*	Low quality, high adherence *N =* 48	*N*	Low quality, low adherence *N =* 64	*N*	Total *N =* 224	*N*	Missing *N =* 81
Primary outcome												
BMI (kg/m^2^)	62	-2.54 (3.07)	41	-1.72 (1.67)	45	-1.99 (2.32)	59	-1.36 (2.39)	207	-1.92 (2.51)	73	-1.57 (2.38)
Secondary outcomes												
Fasting glucose (mL/dL)	62	-4.95 (8.90)	42	-2.13 (8.72)	45	-4.55 (10.14)	59	-2.85 (7.45)	208	-3.70 (8.78)	73	-5.65 (4.93)
Fasting insulin (μU/mL)	62	-3.35 (5.96)	42	-2.39 (8.81)	45	-0.95 (6.00)	59	-4.17 (24.99)	208	-2.87 (14.49)	73	-3.58 (6.47)
SBP (mmHg)	61	-4.85 (9.05)	41	-5.88 (8.29)	45	-3.78 (9.22)	59	-0.33 (8.83)	208	-3.53 (9.07)	73	-2.43 (8.30)
DBP (mmHg)	61	-3.46 (5.52)	41	-3.08 (5.32)	45	-2.86 (5.98)	59	0.27 (4.90)	208	-2.19 (5.60)	73	1.07 (3.12)
Triglycerides (mg/dL)	62	-17.51 (62.03)	42	-18.86 (50.35)	45	-5.44 (43.37)	59	-3.36 (57.90)	208	-11.16 (54.99)	73	-11.06 (68.67)
HDL-C (mg/dL)	62	-1.03 (6.48)	42	0.25 (5.49)	45	-0.19 (5.97)	59	1.67 (5.48)	208	0.18 (5.95)	73	3.75 (5.11)
LDL-C (mg/dL)	62	-6.29 (23.31)	42	2.34 (18.78)	45	-3.87 (18.52)	59	0.42 (17.53)	208	-2.12 (20.02)	73	-8.04 (16.14)
Dietary components												
Energy (kcal)	64	-934.26 (578.64)	48	-132.12 (380.04)	48	-803.98 (449.38)	64	-35.97 (430.93)	224	-477.80 (620.08)	-	-
Carbohydrate (g)	64	-74.80 (79.79)	48	-18.60 (66.48)	48	-50.16 (69.52)	64	-0.81 (68.13)	224	-36.33 (77.18)	-	-
Fat (g)	64	-59.58 (26.84)	48	-7.21 (15.85)	48	-56.42 (23.57)	64	-3.70 (16.88)	224	-31.72 (34.10)	-	-
HEI-2010 Score	64	17.08 (5.85)	48	17.64 (7.22)	48	-1.11 (8.11)	64	-3.92 (8.28)	224	7.30 (12.47)	-	-

Abbreviations: SBP, Systolic Blood Pressure; DBP, Diastolic Blood Pressure; HEI, Healthy Eating Index -2010.

1 Dietary quality is defined by 12-mo minus baseline change in HEI-2010 score (above the median is high, below the median is low).

2 Dietary adherence is defined by 12-mo minus baseline change in grams of fat (below the median is high, above the median is low).

3 All values presented as Mean (SD). N columns are the number of participants whose data was available to include in the analysis of each outcome.

**TABLE 5:** Baseline to 12-mo changes in primary and secondary outcomes for those with high dietary quality, high dietary adherence, or both versus those with low dietary quality and adherence (Change (95% Confidence Interval)).

	High quality, High adherence	High quality, Low adherence	Low quality, High adherence
Primary outcome			
BMI (kg/m^2^)			
Low-carb: M1^1^	-0.64 (-1.48, 0.20)	-0.43 (-1.36, 0.49)	-0.40 (-1.31, 0.52)
Low-carb: M2^2^	-1.15 (-2.04, -0.26)	-0.30 (-1.22, 0.61)	-0.80 (-1.74, 0.14)
Low-fat: M1^1^	-0.98 (-1.88, -0.08)	-0.30 (-1.29, 0.69)	-0.53 (-1.51, 0.44)
Low-fat: M2^3^	-1.11 (-2.10, -0.11)	-0.26 (-1.26, 0.75)	-0.66 (-1.74, 0.41)
Secondary outcomes			
Fasting glucose (mL/dL)			
Low-carb: M1^1^	-4.51 (-7.94, -1.08)	-2.12 (-5.92, 1.68)	-1.46 (-5.25, 2.32)
Low-carb: M2^2^	-5.40 (-9.12, -1.69)	-1.88 (-5.70, 1.93)	-2.12 (-6.04, 1.81)
Low-fat: M1^1^	-1.92 (-5.12, 1.28)	0.72 (-2.79, 4.23)	-1.79 (-5.26, 1.68)
Low-fat: M2^3^	-2.13 (-5.66, 1.40)	0.80 (-2.76, 4.36)	-2.01 (-5.84, 1.81)
Fasting insulin (μU/mL)			
Low-carb: M1^1^	-3.93 (-5.75, -2.10)	-2.93 (-4.95, -0.91)	-1.84 (-3.85, 0.17)
Low-carb: M2^2^	-4.11 (-6.09, -2.13)	-2.88 (-4.92, -0.85)	-1.97 (-4.07, 0.12)
Low-fat: M1^1^	1.21 (-4.04, 6.47)	1.49 (-4.27, 7.26)	4.38 (-1.32, 10.09)
Low-fat: M2^3^	-0.33 (-6.11, 5.44)	2.05 (-3.78, 7.87)	2.73 (-3.52, 8.99)
SBP (mmHg)			
Low-carb: M1^1^	-4.66 (-8.01, -1.31)	-3.32 (-7.03, 0.39)	-4.66 (-8.33, -0.99)
Low-carb: M2^2^	-5.54 (-9.16, -1.92)	-3.09 (-6.81, 0.63)	-5.35 (-9.17, -1.53)
Low-fat: M1^1^	-4.00 (-7.23, -0.77)	-5.61 (-9.16, -2.06)	-2.87 (-6.35, 0.62)
Low-fat: M2^3^	-4.46 (-8.03, -0.90)	-5.45 (-9.04, -1.85)	-3.36 (-7.20, 0.48)
DBP (mmHg)			
Low-carb: M1^1^	-2.40 (-4.59, -0.20)	-1.24 (-3.68, 1.20)	-2.44 (-4.85, -0.03)
Low-carb: M2^2^	-3.04 (-5.42, -0.67)	-1.07 (-3.52, 1.37)	-2.95 (-5.46, -0.44)
Low-fat: M1^1^	-3.67 (-5.66, -1.68)	-3.39 (-5.57, -1.20)	-2.94 (-5.09, -0.79)
Low-fat: M2^3^	-4.09 (-6.28, -1.89)	-3.24 (-5.45, -1.02)	-3.38 (-5.74, -1.01)
Triglycerides (mg/dL)			
Low-carb: M1^1^	-26.88 (-61.34, 7.58)	-8.21 (-46.38, 29.96)	-39.53 (-77.58, -1.49)
Low-carb: M2^2^	-22.62 (-60.04, 14.81)	-9.32 (-47.74, 29.10)	-36.41 (-75.96, 3.14)
Low-fat: M1^1^	-16.77 (-36.80, 3.25)	-15.85 (-37.83, 6.12)	-2.99 (-24.72, 18.74)
Low-fat: M2^3^	-14.65 (-36.75, 7.45)	-16.62 (-38.88, 5.65)	-0.72 (-24.64, 23.20)
HDL-C (mg/dL)			
Low-carb: M1^1^	-0.73 (-3.06, 1.60)	-2.82 (-5.39, -0.24)	-1.10 (-3.69, 1.48)
Low-carb: M2^2^	-1.07 (-3.59, 1.46)	-2.73 (-5.32, -0.14)	-1.34 (-4.02, 1.34)
Low-fat: M1^1^	-2.83 (-4.99, -0.66)	-1.42 (-3.79, 0.96)	-2.03 (-4.38, 0.31)
Low-fat: M2^3^	-2.35 (-4.73, 0.03)	-1.58 (-3.98, 0.81)	-1.53 (-4.11, 1.05)
LDL-C (mg/dL)			
Low-carb: M1^1^	3.81 (-3.44, 11.07)	2.59 (-5.45, 10.63)	0.34 (-7.73, 8.42)
Low-carb: M2^2^	4.17 (-3.72, 12.06)	2.49 (-5.60, 10.59)	0.60 (-7.78, 8.98)
Low-fat: M1^1^	-6.83 (-14.11, 0.44)	1.56 (-6.42, 9.55)	-3.90 (-11.80, 3.99)
Low-fat: M2^3^	-8.20 (-16.23, -0.18)	2.05 (-6.03, 10.13)	-5.37 (-14.05, 3.32)

Results based on linear regression models performed separately for each outcome and for each diet intervention (low-carbohydrate and low-fat), with the low quality, low adherence group as the reference group. Results display parameter estimates and 95% confidence intervals for all models.

Abbreviations: SBP, Systolic Blood Pressure; DBP, Diastolic Blood Pressure.

1 Model 1 adjusted for age (y), sex (male/female), and baseline weight (kg).

2 Model 2 for low carbohydrate adjusted for age (y), sex (male/female), baseline weight (kg), and baseline net carbohydrate intake (g).

3 Model 2 for low fat adjusted for age (y), sex (male/female), baseline weight (kg), and baseline total fat intake (g).

All statistical procedures were performed using SAS (SAS Institute, Cary, NC, version 9.4) and R (R Foundation for Statistical Computing, version 3.3.2), and all statistical tests were evaluated using an alpha cutoff of 0.05.

## Results

Baseline characteristics by dietary quality and adherence to diet are provided in Table 1 for those assigned to an HLC diet and in Table 2 for those assigned to an HLF diet. Across both diet arms, study participants were 56.8% female, with the majority of participants having at least some college education. The racial and ethnic makeup was 58.8% non-Hispanic White, 21.0% Hispanic, 9.9% Asian, and 6.6% other race or ethnicity. Mean baseline weight was 106.2 kg for males and 89.8 kg for females. Mean BMI was 33.4 kg/m^2,^ and mean waist circumference was 112.2 ±9.8 cm for males and 103 ±10.5 cm for females. Mean lipid values were in the normal ranges (LDL <130mg/dL; HDL ≥40mg/dL for males and HDL ≥50 for females; Triglycerides <150mg/dL), and mean fasting glucose concentration was 98.4 mg/dl. Mean HEI-2010 was 55.2 ± 11.7. Mean HEI components at baseline and 12 mo are provided in Supplemental Tables 3 and 4.

There were no missing data at baseline. At 12 mo, 366 participants (81.7%) included in this analysis provided all 3 24-h dietary recalls, 46 provided 2 recalls (10.3%), and 36 (8.0%) provided 1 recall. DIETFITS participants who did not provide dietary recalls at 12 mo were excluded from the analysis (*N =* 161, excluding 80 from HLC and 81 from HLF). Reasons for missing data were the majority (53%) unknown or not provided by participants; other reasons were diet assignment dissatisfaction, health, personal reasons, or a schedule change/move. The number of participants missing data for the clinical measures (i.e., BMI, SBP, DBP, glucose, insulin, triglycerides, HDL cholesterol, LDL cholesterol concentrations) within each quality/adherence subgroup ranged between 1 and 7 (Table 3 and Table 4).

Absolute 12-mo changes in BMI were greatest for HQ/HA and lowest for LQ/LA subgroups for both the HLC and HLF diets; values for HQ/LA and LQ/HA were intermediate between the 2 (Table 3 and Table 4). For the HLC diet group, fasting glucose, insulin concentrations, and SBP followed a similar pattern, as did fasting glucose concentration, DBP, and LDL cholesterol concentration for the HLF diet group. The remainder of clinical measures showed variability between group results for both diet groups (**1**).

**FIGURE 1:**
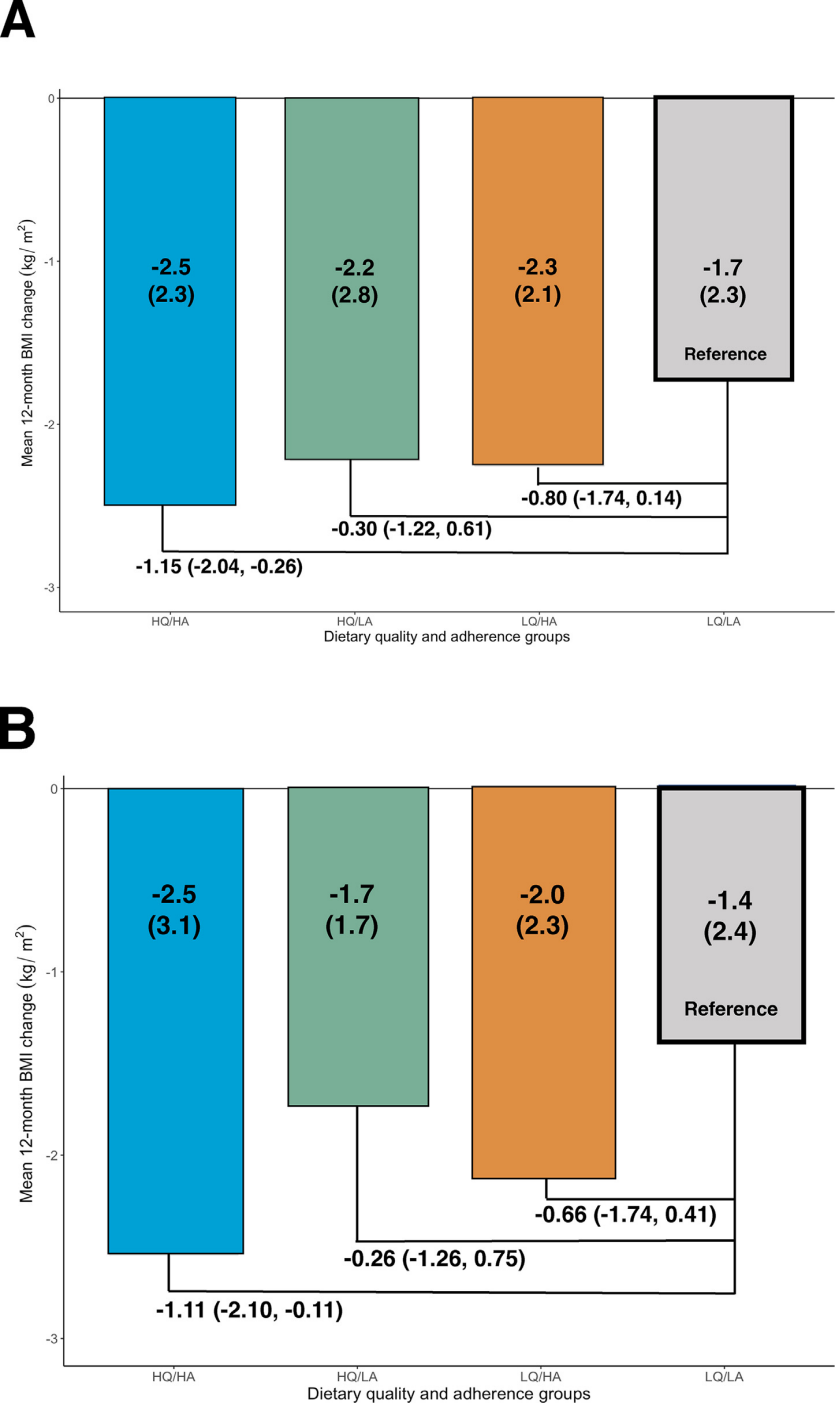
Baseline to 12-mo change in BMI by dietary quality and adherence for those assigned to a healthy low-carbohydrate diet (A) or healthy low-fat diet (B). Dietary quality is defined by 12-mo minus baseline change in HEI-2010 score (above the median is high, below the median is low); dietary adherence is defined by 12-mo minus baseline change in grams of net carbohydrates (below the median is high, above the median is low). Bars display mean (SD) 12-mo BMI change from baseline by diet quality/diet adherence groups. Numbers below the bars represent the changes in mean (95% CI) BMI compared with LQ/LA group, determined from linear regression modeling of 12-mo BMI change on diet quality and adherence groups adjusted for age, sex, baseline weight, and baseline net carbohydrate or total fat intake. Abbreviations: HQ, high quality; HA, high adherence; LQ, low quality; LA, low adherence.

The primary and secondary outcomes were analyzed via multiple linear regression using LQ/LA as the reference group (Table 5). For both the HLC and HLF diets, the HQ/HA subgroups had statistically significant decreases in 12-mo mean BMI [change (95% CI): -1.15 (-2.04, -0.26) for HLC; -1.11 (-2.10, -0.11) for HLF] (Figure 1A,B). The HQ/LA and LQ/HA subgroups also decreased mean BMIs more than the reference group, though these results were not statistically significant. For fasting glucose within HLC, all subgroups improved compared with the reference group, but this was statistically significant only for HQ/HA [-5.40 (-9.12, -1.69)]. For fasting glucose within HLF, only HQ/HA and LQ/HA improved compared with the reference group, though these results were not statistically significant. Fasting insulin values decreased significantly for the HQ/HA subgroup [-4.11 (-6.09, -2.13)] and the HQ/LA [-2.88 (-4.92, -0.85)] within the HLC diet group. Systolic blood pressure decreased significantly for both the HQ/HA [-5.54 (-9.16, -1.92)] and LQ/HA [-5.35 (-9.17, -1.53)] subgroups within the HLC diet group and for both the HQ/HA [-4.46 (-8.03, -0.90)] and HQ/LA [-5.45 (-9.04, -1.85)] subgroups with the HLF diet group. DBP decreased significantly for all subgroups except for HQ/LA within HLC; the greatest decreases were seen for the HQ/HA subgroups [-3.04 (-5.42, -0.67) for HLC; -4.09 (-6.28, -1.89) for HLF]. For triglycerides, values in all subgroups decreased compared with LQ/LA, but the results were not statistically significant. Concentrations of HDL cholesterol were worse for all subgroups compared with LQ/LA, but this was only statistically significant for the HQ/LA subgroup within HLC. Values of LDL cholesterol decreased in HQ/HA and LQ/HA within HLF; however, results were only statistically significant for the HQ/HA subgroup [-8.20 (-16.23, -0.18)]. For sensitivity analysis, adjustment for baseline caloric intake and baseline HEI 2010 score (Supplemental Table 5) did not meaningfully change the results. There were slight changes to lipid values such that those at the border of significance shifted slightly to cross the threshold in both directions.

## Discussion

In this secondary analysis of a randomized controlled weight loss trial for generally healthy adults with overweight and obesity, the participants most successful in reducing their BMIs were those with both higher level of adherence to their assigned diets and higher quality diets. Additionally, this HQ/HA combination was associated with clinically and statistically significant reductions in systolic and diastolic blood pressures for those assigned to follow both HLC and HLF; fasting glucose and fasting insulin concentrations improved only for those assigned to the HLC diet. For the HLC group, HA seemed to drive the associations for both systolic and diastolic blood pressures. For the HLF group, HQ seemed to drive this association for systolic blood pressure; for DBP, either HA or HQ was sufficient to see this significant difference. Also, for the HLF group, HQ was associated with improvements in triglycerides values, and HQ and adherence were associated with slight worsening of HDL cholesterol values.

Within the primary analysis of the DIETFITS trial [11], weight change at 12 mo was −5.3 kg for the HLF diet compared with −6.0 kg for the HLC diet (mean between group difference, 0.7 kg [95% CI, −0.2 to 1.6 kg]). The findings of this post hoc analysis support prior studies showing the importance of adherence to a given dietary strategy for weight loss [[26], [27], [28]]. However, they also highlight the potential added benefits of choosing a high-quality diet within varying dietary strategies—such as low-carbohydrate or low-fat diets—for improved weight loss and blood pressure [[29], [30], [31]]. Much of the research on weight loss diet interventions has focused on a wide variety of dietary changes that directly or indirectly result in reduction of caloric intake [5]. Within the DIETFITS trial [11], calorie restriction was intentionally not part of the intervention but rather a change in the assigned macronutrient (carbohydrate or fat), and improvements in diet quality were the foci. However, in the AHA/ACC/TOS Guideline for Management of Obesity and Overweight in Adults, no explicit mention is made of aiming to improve dietary quality as part of a weight loss diet [5]. Clearly, this is an area that warrants further investigation. There are several scientifically plausible reasons why a higher quality diet may influence weight loss, including higher fiber content, lower calorie density, lower glycemic index, higher water content, and higher nutrient density, among others [[32], [33], [34], [35], [36]]. On a population level in observational studies, those with higher quality diets, as measured by the Healthy Eating Index, also have lower BMIs [[37], [38], [39]].

Moreover, this analysis showed associations between high-quality diets and improvements in metabolic measures such as fasting insulin concentration and systolic and diastolic blood pressures for those following low-fat diets. Additionally, improvements in fasting glucose and insulin values were seen in the low-carbohydrate group only for those with both HQ/HA diet. Improvements in insulin values were seen in the low-carbohydrate group only for both HQ/HA and HQ/LA. Due to the exploratory nature of this analysis, it is possible that these findings were due to chance. However, given the increased risk for metabolic disease among those with overweight and obesity, adding a focus on dietary quality in weight loss dietary interventions in order to potentially reduce risk of, or improve, related conditions independent of weight loss alone is intriguing and supported by the literature [38, [40], [41], [42], [43]].

Patterns of associations between dietary adherence/quality and changes in lipids among those assigned to HLC and HLF diets were less clear. Compared with the LA/LQ subgroup, none of the other adherence/quality groups had significant changes in their LDL cholesterol values. Triglyceride concentrations improved among only the HQ/LA subgroup assigned to the low-fat diet only. HDL cholesterol concentrations worsened very slightly among the HQ/HA subgroup assigned to the low-fat diet. These findings could have been due to a number of reasons, including chance, nondietary factors, or little room to change, given baseline mean lipid values were at or very near goal levels according to current guidelines [44]. Bias toward the null is also a possibility, given the method used to divide participants into dietary adherence/quality subgroups. Changes in dietary quality needed to impact lipids concentrations may also be different than dietary changes made that affected weight loss in this study. When considering lipids values and the dietary adherence/quality relationship, more research is needed.

The DIETFITS trial and this secondary analysis have several strengths. The DIETFITS trial had a large sample size (*N =* 609) that included females and males, had good retention (retention at 12 mo for the DIETFITS main outcomes, which was defined as participants who provided any data at 12 mo, was 79% for both groups), and a sufficient duration (12 mo) to evaluate long-term dietary adherence, dietary quality, and the impacts of these on primary and secondary outcomes. The gold standard for self-reported dietary data collection (i.e., the multipass 24-h recall) was used [45]. Study interventions were created from evidence-based frameworks of behavior change [12, 46, 47]. Medications that can influence weight and metabolism were part of the exclusion criteria; this is important because some of these same medications can also influence food choices. Additionally, baseline characteristics between participants included in the analysis and those with missing data were comparable (Table 1). Because study participants were generally healthy adults with overweight and obesity, it is potentially generalizable to a large segment of the US population. Statistical analysis controlled for baseline fat or carbohydrate intake (Table 5), which would have otherwise influenced the results.

Due to the inherent limitations of a secondary analysis, participants in this study were not randomly assigned to dietary quality/dietary adherence groups, making this essentially an observational study. Another limitation is a likely bias toward the null for all outcomes. This is because the method used to divide participants into diet quality/adherence groups was splitting at the median for 12-mo change in macronutrient (i.e., fat or carbohydrate) intake and median diet quality score (i.e., HEI). Many participants clustered around the median (Supplemental Figures 2A and 2B). To the contrary, a strength of this study is that the statistically significant findings are more likely to be real rather than found due to chance because of this bias. Participants were relatively well-educated compared with the general population and primarily non-Hispanic White, potentially limiting generalizability. A quarter of DIETFITS participants were excluded from this analysis due to missing 12-mo dietary recalls; however, those with missing data were comparable to those included in the analysis (TABLE 1, TABLE 2). Although high dietary quality is associated with better health in the literature [38, [40], [41], [42], [43]], there is no established cutoff above which it is considered high compared with low dietary quality for the outcomes included in the study. Similarly, although greater adherence to assigned diets within a dietary weight loss intervention is associated with greater weight loss [[26], [27], [28]], there is no clear cutoff for determining adherence compared with nonadherence within dietary interventions [48]. This is especially true for DIETFITS as instructions given in the study were to initially reduce to 20 grams of fat or net carbohydrate for 8 wk and then increase intake until a level was reached that participants could maintain long term. This maintenance level varied by participant, and thus, median was chosen as a rational scientific cutoff.

Further research is needed to assess the impact of dietary quality in dietary interventions aimed at weight loss and improvement in cardiovascular disease risk factors. The results of this secondary analysis suggest that rather than focusing solely on changes in macronutrient intake, such as low-carbohydrate and low-fat diets, additional benefits may be gained through emphasizing eating a high-quality diet, regardless of other dietary specifics. However, it should be noted that the quality of the diet is also influenced by macronutrient intake. To date, no studies have shown a negative impact on health of eating a high-quality diet, and countless have shown numerous health benefits. Therefore, in a clinical setting, risk-benefit calculation in deciding whether to recommend high-quality diet regardless of other dietary features is in favor of making this recommendation.

## Acknowledgments

The authors would like to thank the Gardner research team members. Dr. Jennifer Robinson and Antonella Dewell served as study coordinators. Health educators included Rise Cherin, Susan Kirkpatrick, Jae Berman, Dalia Perelman, and Mandy Murphy Carroll. The diet assessment team included Sarah Farzinkhou, Valerie Alaimo, Margaret Shimer, and Diane Demis. Various other important study roles were played by Josephine Lin, Erin Avery, Alexandra Rossi, Katherine Dotter, and Dr. Sarah Mummah. Additional postdoctoral research fellows and visiting scholars who were involved in various phases of the study included Drs. Lisa Offringa, Kenji Nagao, Marily Oppezzo, Ben Chrisinger, and Michael Stanton.

### Author contributions

The authors’ responsibilities were as follows—M.E.H. and J.C.H. formulated the research question and wrote the initial and final drafts of the manuscript, C.D.G designed the DIETFITS research project and contributed to writing both the initial and final drafts of the manuscript, M.J.L. contributed to writing and editing the final drafts, and J.C.H., L.D.G, C.W.S., F.Q., J.R. provided feedback and critical revisions of the manuscript. All authors have read and approved the final manuscript.

### Conflicts of interest

All authors have completed the ICMJE uniform disclosure form and declare: no support from any organization for the submitted work; no financial relationships with any organizations that might have an interest in the submitted work in the previous 3 years; no other relationships or activities that could appear to have influenced the submitted work.

### Funding

National Institute of Diabetes and Digestive and Kidney Diseases, NIH 1R01DK091831; Nutrition Science Initiative (NuSI); National Heart, Lung, and Blood InstituteNIH T32HL007034; NIH 1 K12 GM088033; Stanford Clinical and Translational Science Award (CTSA). The content is solely the responsibility of the authors and does not necessarily represent the official views of the NIH or other funders.

The study funders had no role in the study design, in the collection, analysis, and interpretation of data, in the writing of the report, or in the decision to submit the article for publication. All authors, external and internal, had full access to all the data (including statistical reports and tables) in the study and can take responsibility for the integrity of the data, and the accuracy of the data analysis is also required.

### Data availability

The data that support the findings of this study are available from the corresponding author, C.D.G., upon reasonable request.

## Appendix A Supplementary data

Multimedia component1 Multimedia component1

Multimedia component2 Multimedia component2
