# Supplementary material for: Association of dietary adherence and dietary quality with weight loss success among those following low-carbohydrate and low-fat diets: a secondary analysis of the DIETFITS randomized clinical trial
Source: Am J Clin Nutr. 2023 Nov 4;119(1):174–84. doi: 10.1016/j.ajcnut.2023.10.028 (PMC10808819; doi:10.1016/j.ajcnut.2023.10.028)
Supplement: Multimedia component1 [file mmc1.pptx]

## Slide 1
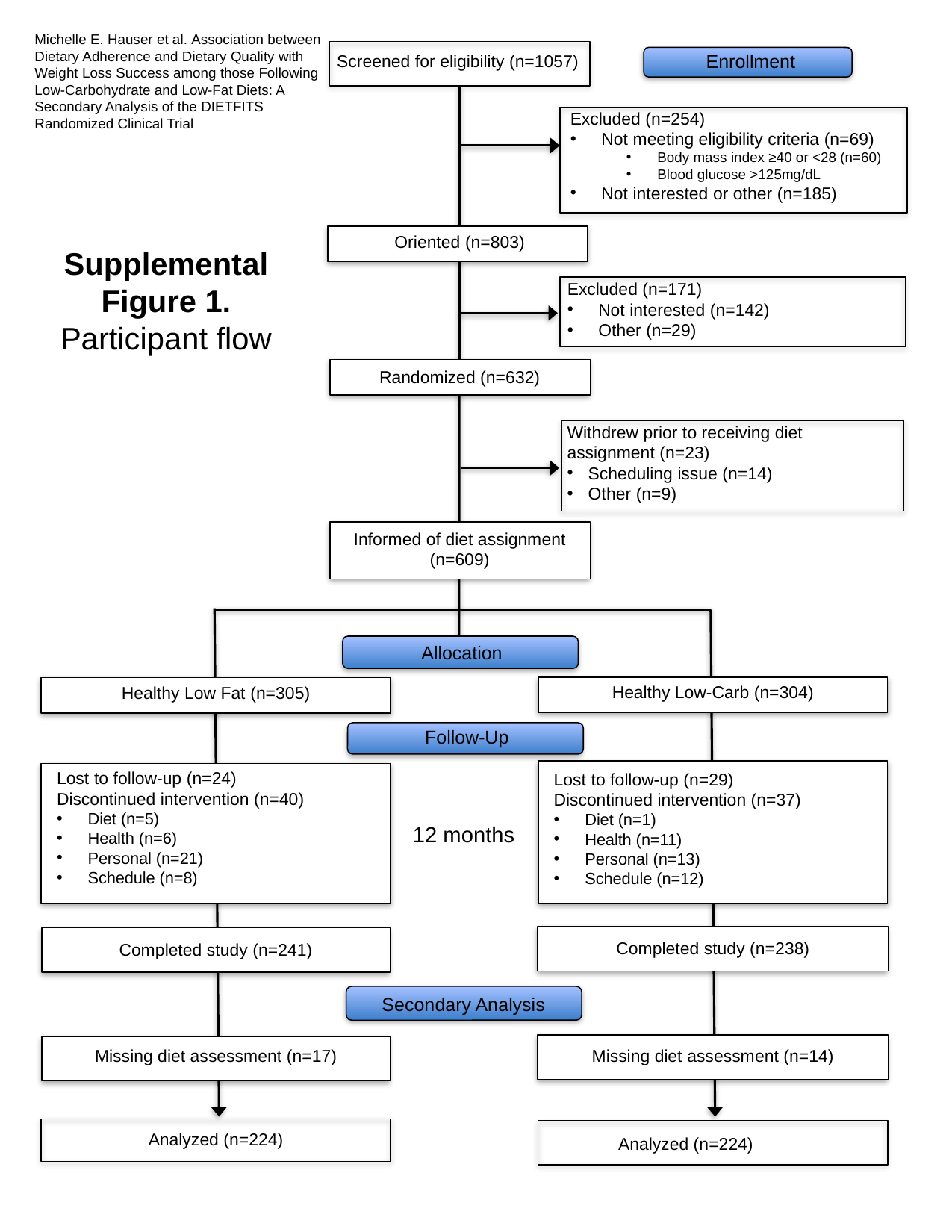

Michelle E. Hauser et al. Association between Dietary Adherence and Dietary Quality with Weight Loss Success among those Following Low-Carbohydrate and Low-Fat Diets: A Secondary Analysis of the DIETFITS Randomized Clinical Trial
Enrollment
Screened for eligibility (n=1057)
Excluded (n=254)
Not meeting eligibility criteria (n=69)
Body mass index ≥40 or <28 (n=60)
Blood glucose >125mg/dL
Not interested or other (n=185)
Oriented (n=803)
Supplemental Figure 1. Participant flow
Excluded (n=171)
Not interested (n=142)
Other (n=29)
Randomized (n=632)
Withdrew prior to receiving diet assignment (n=23)
Scheduling issue (n=14)
Other (n=9)
Informed of diet assignment (n=609)
Allocation
Healthy Low-Carb (n=304)
Healthy Low Fat (n=305)
Follow-Up
Lost to follow-up (n=24)
Discontinued intervention (n=40)
Diet (n=5)
Health (n=6)
Personal (n=21)
Schedule (n=8)
Lost to follow-up (n=29)
Discontinued intervention (n=37)
Diet (n=1)
Health (n=11)
Personal (n=13)
Schedule (n=12)
12 months
Completed study (n=238)
Completed study (n=241)
Secondary Analysis
Missing diet assessment (n=14)
Missing diet assessment (n=17)
Analyzed (n=224)
Analyzed (n=224)
