# Supplementary material for: Association of dietary adherence and dietary quality with weight loss success among those following low-carbohydrate and low-fat diets: a secondary analysis of the DIETFITS randomized clinical trial
Source: Am J Clin Nutr. 2023 Nov 4;119(1):174–84. doi: 10.1016/j.ajcnut.2023.10.028 (PMC10808819; doi:10.1016/j.ajcnut.2023.10.028)
Supplement: Multimedia component2 [file mmc2.docx]

**Online Supplemental Material**

**Association of Dietary Adherence and Dietary Quality with Weight Loss Success among those Following Low-Carbohydrate and Low-Fat Diets: A Secondary Analysis of the DIETFITS Randomized Clinical Trial**

Michelle E. Hauser, Jennifer C. Hartle, Matthew J. Landry, Priya Fielding-Singh, Cynthia W. Shih, FeiFei Qin, Joseph Rigdon, Christopher D. Gardner

| **Supplemental Table 1.** Median 12-Month Change in HEI-2010 and Dietary Carbohydrate for those Assigned to a Healthy Low-Carbohydrate Diet (n=244)^1^ | | |
| --- | --- | --- |
|  | **Median 12-month HEI-2010 Change** | **Median 12-month Change in Carbohydrate (g) Intake** |
| High Adherence | 8.30 | -159.46 |
| Low Adherence | 0.51 | -59.19 |
| High Quality | 12.81 | -119.01 |
| Low Quality | -3.88 | -91.63 |
| ^1^The HEI-2010 is made up of 12 components, nine of the components focus on adequacy (dietary components to increase) and three (refined grains, sodium, and empty calories) focus on moderation (dietary components to decrease). All components have a maximum score of 5 with the exception of whole grains, dairy, fatty acids, refined grains, and sodium having a score of 10 and empty calories having a maximum score of 20. The 12 components sum to a maximum total score of 100 and signifies the highest possible compliance to the 2010 Dietary Guidelines for Americans.  Abbreviations: HEI, Healthy Eating Index | | |

| **Supplemental Table 2.** Median 12-Month Change in HEI-2010 and Dietary Fat for those Assigned to a Healthy Low-Fat Diet (n=244)^1^ | | |
| --- | --- | --- |
|  | **Median 12-month HEI-2010 Change** | **Median 12-month Change in Fat (g) Intake** |
| High Adherence | 10.60 | -51.41 |
| Low Adherence | 4.57 | -7.30 |
| High Quality | 16.19 | -35.74 |
| Low Quality | -1.28 | -23.37 |
| ^1^The HEI-2010 is made up of 12 components, nine of the components focus on adequacy (dietary components to increase) and three (refined grains, sodium, and empty calories) focus on moderation (dietary components to decrease). All components have a maximum score of 5 with the exception of whole grains, dairy, fatty acids, refined grains, and sodium having a score of 10 and empty calories having a maximum score of 20. The 12 components sum to a maximum total score of 100 and signifies the highest possible compliance to the 2010 Dietary Guidelines for Americans.  Abbreviations: HEI, Healthy Eating Index | | |

| **Supplemental Table 3**. Healthy Eating Index-2010 (HEI) component scores (Mean (SD)) by dietary quality and adherence to diet for those assigned to a healthy low-carbohydrate diet ^1,2^ | | | | | | |
| --- | --- | --- | --- | --- | --- | --- |
| **HEI-2010 Component** | **High quality, high adherence**  **(HQ/HA)**  **N=67** | **High quality, low adherence**  **(HQ/LA)**  **N=45** | **Low quality, high adherence**  **(LQ/HA)**  **N=45** | **Low quality, low adherence**  **(LQ/LA)**  **N=67** | **Total**  **N=224** | **Missing**  **N=80** |
| **Baseline (n=304)** | | | | | | |
| Total Protein Foods | 4.31 (0.79) | 4.52 (0.70) | 4.46 (0.70) | 4.63 (0.51) | 4.48 (0.69) | 4.42 (0.73) |
| Greens and Beans | 2.15 (1.38) | 2.11 (1.68) | 3.01 (1.50) | 2.75 (1.60) | 2.49 (1.57) | 2.16 (1.67) |
| Seafood and Plant Proteins | 1.91 (1.31) | 2.28 (1.75) | 3.14 (1.45) | 2.89 (1.27) | 2.52 (1.50) | 2.14 (1.40) |
| Total Fruit | 1.53 (1.35) | 1.45 (1.16) | 2.36 (1.26) | 2.47 (1.47) | 1.96 (1.41) | 1.72 (1.31) |
| Whole Fruit | 1.67 (1.66) | 1.86 (1.46) | 2.79 (1.49) | 2.93 (1.62) | 2.31 (1.67) | 2.07 (1.53) |
| Total Vegetables | 2.97 (1.08) | 3.26 (1.16) | 3.8 (0.89) | 3.59 (1.23) | 3.38 (1.15) | 3.26 (1.22) |
| Whole Grain | 3.51 (2.36) | 3.30 (2.80) | 4.97 (2.83) | 4.09 (2.59) | 3.93 (2.67) | 3.62 (2.56) |
| Dairy | 5.5 (2.55) | 4.81 (2.74) | 5.16 (2.30) | 5.06 (2.71) | 5.16 (2.59) | 5.17 (2.41) |
| Fatty Acids | 4.16 (2.28) | 4.76 (2.42) | 5.36 (2.86) | 5.96 (2.32) | 5.06 (2.53) | 4.68 (2.41) |
| Refined Grains^3^ | 5.47 (2.28) | 6.22 (2.28) | 7.48 (2.23) | 7.48 (2.12) | 6.63 (2.38) | 6.25 (2.43) |
| Sodium^3^ | 4.29 (2.46) | 3.62 (2.21) | 5.46 (2.56) | 4.52 (2.77) | 4.46 (2.58) | 4.62 (2.86) |
| Empty Calories^3^ | 11.24 (4.12) | 13.04 (4.22) | 13.90 (4.00) | 14.88 (4.29) | 13.23 (4.38) | 12.98 (4.09) |
| **12 months (n=244)** | | | | | | |
| Total Protein Foods | 4.94 (0.33) | 4.89 (0.3) | 4.95 (0.21) | 4.80 (0.36) | 4.89 (0.32) | - |
| Greens and Beans | 3.86 (1.32) | 3.27 (1.69) | 3.10 (1.8) | 3.15 (1.77) | 3.38 (1.66) | - |
| Seafood and Plant Proteins | 3.47 (1.47) | 3.75 (1.24) | 3.06 (1.74) | 2.91 (1.64) | 3.28 (1.56) | - |
| Total Fruit | 2.03 (1.55) | 2.09 (1.59) | 1.47 (1.3) | 1.70 (1.42) | 1.83 (1.48) | - |
| Whole Fruit | 2.65 (1.73) | 2.57 (1.87) | 1.95 (1.69) | 2.04 (1.49) | 2.31 (1.70) | - |
| Total Vegetables | 4.38 (0.93) | 4.01 (0.91) | 4.11 (1.24) | 4.00 (1.19) | 4.14 (1.08) | - |
| Whole Grain | 2.09 (2.67) | 3.38 (3.3) | 1.17 (2.13) | 2.27 (2.56) | 2.22 (2.76) | - |
| Dairy | 6.04 (2.46) | 5.03 (2.28) | 5.77 (2.80) | 5.57 (2.59) | 5.64 (2.54) | - |
| Fatty Acids | 5.58 (2.51) | 6.62 (2.29) | 4.34 (2.84) | 4.29 (2.42) | 5.15 (2.66) | - |
| Refined Grains^3^ | 9.13 (1.45) | 8.90 (1.67) | 9.12 (1.53) | 8.06 (2.02) | 8.76 (1.75) | - |
| Sodium^3^ | 3.79 (2.68) | 4.55 (2.9) | 3.51 (2.83) | 3.57 (2.59) | 3.82 (2.74) | - |
| Empty Calories^3^ | 15.92 (3.49) | 15.55 (3.47) | 14.01 (4.89) | 12.45 (4.77) | 14.42 (4.43) | - |
| ^1^ The HEI-2010 is made up of 12 components, nine of the components focus on adequacy (dietary components to increase) and three (refined grains, sodium, and empty calories) focus on moderation (dietary components to decrease). All components have a maximum score of 5 except for whole grains, dairy, fatty acids, refined grains, and sodium having a score of 10 and empty calories having a maximum score of 20.  ^2^ The 12 components sum to a maximum total score of 100 and signifies the highest possible compliance to the 2010 Dietary Guidelines for Americans. Because USDA food pattern recommendations for amounts of food groups, oils, and empty calories are stated in terms of absolute amounts that vary according to energy level, the HEI–2010 scores use standards that are expressed as either a percent of calories or per 1,000 calories. This "density" approach uncouples diet quality from quantity. The one exception is fatty acids, which are expressed as a ratio of unsaturated fatty acids to saturated fatty acids.  ^3^ For moderation components, a reverse scoring is applied meaning that a higher score indicates lower consumption. | | | | | | |

| **Supplemental Table 4**. Healthy Eating Index-2010 (HEI) component scores (Mean (SD)) by dietary quality and adherence to diet for those assigned to a healthy low-fat diet ^1,2^ | | | | | | |
| --- | --- | --- | --- | --- | --- | --- |
| **HEI-2010 Component** | **High quality, high adherence**  **(HQ/HA)**  **N=64** | **High quality, low adherence**  **(HQ/LA)**  **N=48** | **Low quality, high adherence**  **(LQ/HA)**  **N=48** | **Low quality, low adherence**  **(LQ/LA)**  **N=64** | **Total**  **N=224** | **Missing**  **N=81** |
| **Baseline (n=305)** | | | | | | |
| Total Protein Foods | 4.26 (0.75) | 4.28 (0.85) | 4.34 (0.83) | 4.47 (0.74) | 4.34 (0.79) | 4.42 (0.72) |
| Greens and Beans | 2.30 (1.39) | 2.25 (1.62) | 2.73 (1.68) | 3.07 (1.37) | 2.60 (1.53) | 2.29 (1.58) |
| Seafood and Plant Proteins | 2.38 (1.55) | 2.24 (1.51) | 2.94 (1.41) | 3.02 (1.42) | 2.65 (1.50) | 2.48 (1.58) |
| Total Fruit | 1.95 (1.49) | 1.81 (1.27) | 1.29 (1.05) | 2.31 (1.37) | 1.88 (1.37) | 1.61 (1.49) |
| Whole Fruit | 2.30 (1.69) | 2.10 (1.53) | 2.00 (1.56) | 2.63 (1.45) | 2.28 (1.57) | 1.86 (1.68) |
| Total Vegetables | 3.16 (1.13) | 3.24 (1.24) | 3.48 (1.15) | 3.88 (1.08) | 3.45 (1.17) | 3.32 (1.19) |
| Whole Grain | 3.85 (2.76) | 3.83 (3.11) | 4.05 (2.81) | 5.04 (3.01) | 4.23 (2.95) | 3.10 (2.78) |
| Refined Grains | 6.39 (2.44) | 5.87 (2.77) | 7.04 (2.27) | 7.07 (2.56) | 6.61 (2.54) | 6.71 (2.62) |
| Dairy | 5.35 (2.68) | 5.67 (2.34) | 6.03 (2.37) | 5.29 (2.47) | 5.55 (2.49) | 4.67 (2.69) |
| Fatty Acids | 4.53 (2.61) | 4.66 (2.36) | 5.72 (2.7) | 5.58 (2.64) | 5.11 (2.62) | 4.59 (2.44) |
| Sodium | 3.89 (2.25) | 3.90 (2.51) | 4.89 (2.55) | 4.26 (2.53) | 4.21 (2.47) | 4.41 (2.24) |
| Empty Calories | 12.46 (4.38) | 13.67 (4.3) | 14.28 (3.85) | 14.62 (4.43) | 13.73 (4.33) | 2.95 (4.51) |
| **12 months (n=244)** | | | | | | |
| Total Protein Foods | 4.44 (0.81) | 4.63 (0.73) | 4.29 (1.06) | 4.28 (0.97) | 4.40 (0.90) | - |
| Greens and Beans | 3.08 (1.65) | 3.84 (1.37) | 2.64 (1.70) | 2.54 (1.66) | 2.99 (1.67) | - |
| Seafood and Plant Proteins | 2.70 (1.62) | 3.37 (1.61) | 2.07 (1.30) | 2.40 (1.74) | 2.62 (1.64) | - |
| Total Fruit | 3.11 (1.5) | 2.97 (1.50) | 1.86 (1.45) | 2.19 (1.49) | 2.55 (1.56) | - |
| Whole Fruit | 3.56 (1.56) | 3.64 (1.37) | 2.3 (1.65) | 2.61 (1.74) | 3.04 (1.68) | - |
| Total Vegetables | 3.97 (1.09) | 4.44 (0.81) | 3.78 (1.03) | 3.85 (1.08) | 4.00 (1.04) | - |
| Whole Grain | 7.15 (2.55) | 5.03 (3.42) | 5.47 (3.29) | 4.20 (3.17) | 5.49 (3.28) | - |
| Dairy | 5.65 (3.02) | 5.82 (2.88) | 5.16 (3.10) | 5.72 (2.42) | 5.60 (2.84) | - |
| Fatty Acids | 6.27 (2.66) | 7.08 (2.20) | 4.74 (2.54) | 4.74 (2.86) | 5.68 (2.77) | - |
| Refined Grains^3^ | 7.81 (1.95) | 7.99 (2.25) | 6.33 (2.80) | 6.22 (2.81) | 7.08 (2.59) | - |
| Sodium^3^ | 4.77 (2.98) | 4.67 (2.95) | 3.28 (2.47) | 4.09 (2.37) | 4.23 (2.75) | - |
| Empty Calories^3^ | 17.40 (3.42) | 17.68 (3.02) | 15.77 (3.33) | 14.46 (4.49) | 16.27 (3.88) | - |
| ^1^ The HEI-2010 is made up of 12 components, nine of the components focus on adequacy (dietary components to increase) and three (refined grains, sodium, and empty calories) focus on moderation (dietary components to decrease). All components have a maximum score of 5 except for whole grains, dairy, fatty acids, refined grains, and sodium having a score of 10 and empty calories having a maximum score of 20.  ^2^ The 12 components sum to a maximum total score of 100 and signifies the highest possible compliance to the 2010 Dietary Guidelines for Americans. Because USDA food pattern recommendations for amounts of food groups, oils, and empty calories are stated in terms of absolute amounts that vary according to energy level, the HEI–2010 scores use standards that are expressed as either a percent of calories or per 1,000 calories. This "density" approach uncouples diet quality from quantity. The one exception is fatty acids, which are expressed as a ratio of unsaturated fatty acids to saturated fatty acids.  ^3^ For moderation components, a reverse scoring is applied meaning that a higher score indicates lower consumption. | | | | | | |

| **Supplemental Table 5:** Baseline to 12-month changes in primary and secondary outcomes for those with high dietary quality, high dietary adherence or both versus those with low dietary quality and adherence (Change (95% Confidence Interval)) | | | |
| --- | --- | --- | --- |
|  | **High quality,**  **High adherence** | **High quality,**  **Low adherence** | **Low quality, High adherence** |
| Primary outcome |  |  |  |
| BMI (kg/m^2^) |  |  |  |
| Low-carb: M3^1^ | -1.06 (-2.03, -0.09) | -0.21 (-1.19, 0.77) | -0.82 (-1.76, 0.13) |
| Low-fat: M3^2^ | -1.27 (-2.30, -0.25) | -0.50 (-1.55, 0.56) | -0.69 (-1.76, 0.39) |
| Secondary outcomes |  |  |  |
| Fasting glucose (mL/dL) |  |  |  |
| Low-carb: M3^1^ | -6.48 (-10.49, -2.46) | -2.97 (-7.03, 1.08) | -1.89 (-5.82, 2.04) |
| Low-fat: M3^2^ | -2.11 (-5.78, 1.57) | 1.03 (-2.74, 4.79) | -1.99 (-5.83, 1.85) |
| Fasting insulin (μU/mL) |  |  |  |
| Low-carb: M3^1^ | -3.89 (-6.04, -1.74) | -2.60 (-4.77, -0.43) | -2.03 (-4.14, 0.07) |
| Low-fat: M3^2^ | 0.36 (-5.48, 6.20) | 0.91 (-5.08, 6.90) | 2.59 (-3.51, 8.69) |
| SBP (mmHg) |  |  |  |
| Low-carb: M3^1^ | -5.70 (-9.64, -1.75) | -3.21 (-7.20, 0.78) | -5.32 (-9.18, -1.47) |
| Low-fat: M3^2^ | -5.06 (-8.76, -1.37) | -6.10 (-9.88, -2.33) | -3.42 (-7.27, 0.43) |
| DBP (mmHg) |  |  |  |
| Low-carb: M3^1^ | -2.90 (-5.49, -0.31) | -0.96 (-3.57, 1.66) | -2.98 (-5.50, -0.45) |
| Low-fat: M3^2^ | -4.51 (-6.78, -2.25) | -3.89 (-6.20, -1.57) | -3.44 (-5.80, -1.08) |
| Triglycerides (mg/dL) |  |  |  |
| Low-carb: M3^1^ | -33.92 (-74.34, 6.51) | -17.31 (-58.13, 23.52) | -34.87 (-74.4, 4.66) |
| Low-fat: M3^2^ | -21.78 (-44.37, 0.81) | -26.67 (-49.84, -3.50) | -1.71 (-25.33, 21.91) |
| HDL-C (mg/dL) |  |  |  |
| Low-carb: M3^1^ | -1.03 (-3.78, 1.72) | -2.75 (-5.53, 0.03) | -1.33 (-4.03, 1.37) |
| Low-fat: M3^2^ | -2.60 (-5.08, -0.13) | -1.90 (-4.44, 0.64) | -1.56 (-4.15, 1.03) |
| LDL-C (mg/dL) |  |  |  |
| Low-carb: M3^1^ | 2.72 (-5.85, 11.29) | 1.27 (-7.39, 9.93) | 0.80 (-7.62, 9.22) |
| Low-fat: M3^2^ | -7.01 (-15.34, 1.31) | 3.03 (-5.51, 11.57) | -5.28 (-13.98, 3.42) |
| Results based on linear regression models performed separately for each outcome and for each diet intervention (low-carbohydrate and low-fat) with the low quality, low adherence group as the reference group. Results display parameter estimates and 95% confidence intervals for all models.  ^1^Model 3 for low carbohydrate adjusted for age (years), sex (male/female), baseline weight (kg), baseline net carbohydrate intake (g), baseline HEI-2010, and baseline calories (energy, kcals)  ^2^Model 3 for low fat adjusted for age (years), sex (male/female), baseline weight (kg), baseline total fat intake (g), baseline HEI-2010, and baseline calories (energy, kcals)  Abbreviations: SBP, Systolic Blood Pressure; DBP, Diastolic Blood Pressure; HDL-C, High-density Lipoprotein Cholesterol; LDL-C, Low-density Lipoprotein Cholesterol | | | |

| **A** |  | **B** |  |
| --- | --- | --- | --- |

**Supplemental Figure 2. (A)** 12-month change in Healthy Eating Index-2010 and grams of carbohydrate. (**B)** 12-month change in Healthy Eating Index-2010 and grams of fat.
